# Concurrent Lactation and Pregnancy: Pregnant Domestic Horse Mares Do Not Increase Mother-Offspring Conflict during Intensive Lactation

**DOI:** 10.1371/journal.pone.0022068

**Published:** 2011-08-10

**Authors:** Jitka Bartošová, Martina Komárková, Jana Dubcová, Luděk Bartoš, Jan Pluháček

**Affiliations:** 1 Department of Ethology, Institute of Animal Science, Praha – Uhříněves, Czech Republic; 2 Department of Zoology, Faculty of Science, Charles University in Prague, Praha, Czech Republic; Texas A&M University, United States of America

## Abstract

Lactation is the most energy demanding part of parental care in mammals, so parent-offspring conflict arises over milk provided by the mother. In some species females commonly become pregnant shortly after parturition of previous young. This further intensifies mother-offspring conflict due to concurrent pregnancy and lactation. In equids it has been well established that pregnant females wean their foals earlier than non-pregnant ones. Intensified mother-offspring conflict was presumed to associate with pregnancy also during the period of intensive lactation, i.e., before the weaning process starts. We investigated the effect of pregnancy on suckling behaviour characteristics as indicators of mother-offspring conflict in domestic horses. Contrary to expectation, here we provide evidence of a decreased mother-offspring conflict related to pregnancy in lactating females during first two trimesters of pregnancy. Pregnant mares provided longer suckling bouts and did not reject or terminate suckling of their foals more often than non-pregnant mares. Our results suggest that pregnant mares cope with parallel investment into a nursed foal and a foetus through enhancing nursing behaviour in early stages of pregnancy before the initially low requirements of the foetus increase. They compensate their suckling foal with the perspective of its early weaning due to ongoing pregnancy.

## Introduction

Among mammals, lactation is the most energy demanding part of parental care [1], so parent-offspring conflict [2] is expected over milk provided by the mother. In particular, parent and offspring should disagree over how long the period of the parental investment should last and over the amount of parental investment that should be provided [2].

In some species, females commonly become pregnant shortly after parturition of previous young, which further intensifies mother-offspring conflict due to concurrent pregnancy and intensive lactation, e.g., in many small rodents and lagomorphs [3]–[5] or some ungulate species, including equids [6]–[8]. In horses, lactating mares under natural conditions usually get pregnant again during the first or second oestrus after giving birth (e.g., [6], [7], [9] post-partum mares experience oestrus, commonly called “foal heat”, during the first week after birth [9].

The mares of free living horses [6], [10], [11] follow the general assumption that the optimum weaning time for pregnant mothers conceiving shortly after giving birth should be earlier than for non-pregnant ones [12]. Earlier weaning in pregnant mares was apparent even in plains zebra mares kept in captivity under optimal nutritional conditions [13]. Females of equid species time weaning according to the expected date of next delivery [7], [11], [13], [14].

While these studies demonstrate well the crucial impact of pregnancy in lactating equid female on mother-offspring conflict over the period of the parental investment (i.e., weaning conflict), little is known about how mother's pregnancy influences conflict between the mother and her offspring over the amount of parental investment she provides during lactation. It was found only recently that in captive plains zebra there are shorter suckling bouts in pregnant mares than non-pregnant ones, suggesting higher mother-offspring conflict when the mother was pregnant [15].

Although suckling behaviour was not found a reliable indicator of milk or energy intake [16], even in foals [17], it can be considered as the behavioural measure associated with parent-offspring conflict over maternal resources [16], [17] and as the measure of the level of maternal care provided by the mother in contrast with foal's needs [10], [18], [19]. Termination of suckling seems to be of great importance. Suckling bouts terminated by the mother are usually shorter compared to those terminated by the foal [15], [20]. As such, it probably indicates that the foal was not satiated [17].

In the present study, we tested effect of pregnancy on suckling behaviour variables as indicators of mother-offspring conflict in domestic horses. We hypothesised shorter suckling bouts and higher rates of rejected and/or terminated suckling in pregnant mares compared to non-pregnant ones caused by increased conflict over amount of maternal investment between mother and her nursed young are to be expected because of her parallel investment into two offspring, a nursed foal and a foetus.

## Methods

### Ethics statement

This study received approval for animal use and care from The Institutional Animal Care and Use Committee of the Institute of Animal Science and was conducted in accordance with Czech Central Committee for Protection of Animals number 13803/2003-1020.

### Subjects and study site

The studied animals were eight groups of loose housed lactating mares with foals of Kladruby horse at the National Stud Kladruby nad Labem, Czech Republic (50°3′29″N, 15°29′4.998″E). Each group containing from 8 to 14 mares was housed in a barn of 10×35 m. Within two seasons, 59 mares (aged from 4 to 25 years, 24 being primiparous) gave birth to 79 foals (32 males, 47 females). In 41 cases (51.9%), a nursing mare became pregnant during lactation.

The mares gave birth to their foals freely in the barn. Animals from all groups were joined and pastured together daily from 9:00 h a.m. to 3:00 h p.m. The mares were fed with oat, minerals and vitamins in the morning and hay was served twice a day. The foals had free access to the food served to their mothers. They started to intake oat and hay individually between two weeks and two months after birth. Horses had permanent ad-libitum access to water. Although the equine reproduction under domestic condition is far from natural conditions as it is completely managed by man, common practice usually follows biological patterns that the mares are bred within the first two post-partum oestrus cycles which was also our case; 75% of the mares became pregnant again within the first or second post-partum oestrus. The mares conceived from 7 to 115 days after previous delivery (mean = 29.8±30.4, median = 10, modus = 9 days; N = 41).

### Data collection

The groups of mares and foals were observed from deliveries to abrupt weaning (ranged from four to seven months of foals' age). Hence the time a particular foal was observed for differed according to its date of birth, group and time of weaning. Each of the foals was observed for at least 127 days and for no more than 210 days.

The method of observation was designed according to previous studies [17], [21]–[23]. Each group was observed in the barn once a fortnight in two three-hour sessions; from 3.30 a.m. to 6.30 a.m. and from 3 p.m. to 6 p.m. Four people were trained for observing horse behaviour. At the beginning of this study, we tested the interobserver reliability in recognizing the recorded behaviours using kappa statistics. Since the behavioural elements observed were simple and easily distinguishable, the reliability was high and, therefore, we did not check this in the later stages of the study. The ad libitum sampling method [24] was applied to catch almost all suckling occurring in the herd. Horses were accustomed to frequent presence of people.

Each of the observed suckling events in the group (called ‘suckling solicitations’) was referred either as ‘rejected sucking attempt’ (i.e., the foal puts its nose near to mother's udder, but does not contact it or suckles for less than 5 s) or a ‘suckling bout’ (contact with the udder lasted 5 s or more). The suckling bout was considered to start at the moment when the foal took the mother's teat in its lips. A new suckling bout or sucking attempt was recorded when the foal lost the contact with the teat for longer than 60 s [17], [25]. Information about mare's birth date and number of previous foals was acquired from the stud archive.

### Statistical analyses

The statistical analyses were performed using The SAS System for Windows 9.2 (SAS). We tested the effect of mare's pregnancy (yes/no) on five suckling characteristics: rate of suckling solicitations rejected by the mother, rate of suckling bouts terminated by the mother, suckling bout duration, frequency of suckling solicitations, and frequency of suckling bouts. All models included also other factors with potential influence on suckling behaviour, i.e., age and sex of the foal, mother's age and parity (primiparous/multiparous), identity of the group (1–8), season (2005/2006) and relevant first term interactions, which are mostly not discussed further in this study.

In case of suckling frequencies and suckling bout duration, multivariate general linear mixed models (GLMM, PROC MIXED, SAS) treated with repeated measures on a particular individual (RANDOM statement) were fitted. The significance of each fixed effect in the GLMM was assessed using *F* - test. Within group least squares means were appropriately adjusted for the other factors in the model with unbalanced design and for multiple comparisons between classes (Tukey–Kramer adjustment).

Frequencies of suckling solicitations (i.e., variable reflecting foals' suckling requirements) and frequency of suckling bouts (i.e., numbers of suckling allowed by the mother) were scored as the total numbers of suckling solicitations or suckling bouts recorded during each observation day (i.e., within the two three-hour sessions) for each individual foal.

Relationship between suckling frequency and duration (expressed as mean suckling bout duration per observation day) was tested using Spearman partial rank-order correlation (PROC CORR, SAS; partial variable: age of the foal).

Probabilities that a mother rejects a suckling solicitation (i.e., suckling is not allowed) or terminates a suckling bout were analyzed by a generalized linear model (GzLM) for categorical data analysis based on the generalized estimating equation approach processed in the GENMOD procedure (binomial distribution, logit link function; repeated measures on the same individuals treated with the REPEATED statement).

Suckling bouts terminated other than by a mother or a foal (e.g., by another animal or external disturbance) did not enter the analyses with exception of suckling frequencies.

The effect of nursing a previous foal or not during mare's pregnancy on birth weight of her foal was tested by general linear model (PROC MIXED, SAS) to control for possible negative effects of lactation on the foetus. Birth weight of the foals born within the season following observations ranged from 41 to 80 kg (mean = 65.3±6.3 kg, N = 74).

## Results

Within 516 hours of observation of 79 mare-foal pairs we recorded 10 848 suckling solicitations, from which 10 607 resulted in a suckling bout. The mothers terminated 14.96% of suckling bouts. Suckling bout duration ranged between 5 and 198 s (mean ± SE: 73.8±28.9 s). Suckling bouts terminated by the mother were considerably shorter than those the foal terminated itself (62.96±0.89 s vs. 76.93±0.61 s, F_(1, 9776)_ = 319.6, P<0.0001, GLMM, PROC MIXED, SAS).

We found no significant effect of pregnancy on probability of the mother rejecting suckling solicitation of her foal (n. s., GzLM, PROC GENMOD, SAS). Nevertheless, sucking attempts rejected by the mother accounted just for 1.0% of observed suckling solicitations and the predicted probability did not exceed 0.028 so they did not seem to be generally of great importance in our case. There was also no significant effect of pregnancy of the mother on the probability of her terminating a suckling bout (n. s., GzLM, PROC GENMOD, SAS).

Neither frequency of suckling solicitations (13.2±5.11, mean ± SD, ranging from 1 to 40 suckling solicitations per observation day) nor suckling bouts (13.0±5.01, ranging from 1 to 38 suckling bouts per observation day) were influenced by reproductive status of the mother (n.s., GLMM, PROC MIXED, SAS).

**Figure 1:**
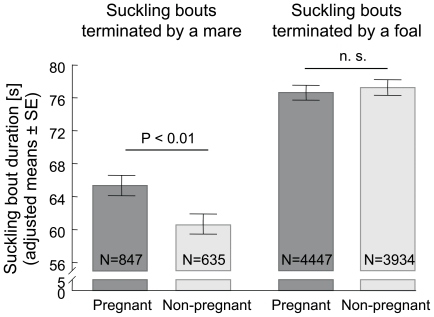
Suckling bout duration [s] according to the reproductive status of the mother when suckling bouts terminated the mother (left) or the foal (right). (Differences between categories including different terminator of a suckling bout were all P<0.0001.)

However the overall effect of mother's pregnancy on suckling bout duration was not significant (70.99 s in pregnant vs. 68.90 s in non-pregnant mares, n. s., GLMM, PROC MIXED, SAS), there were considerable differences in pregnant and non-pregnant mares when taking into account who terminated a suckling bout, whether the mother or the foal (interaction pregnancy*terminator: F_(1, 9776)_ = 12.1, P<0.001). In case of the mother terminated a suckling bout the suckling bouts were longer in pregnant (65.36±1.25 s) than in non-pregnant mares (60.55±1.36 s) while there was no impact detected of pregnancy on duration of suckling bouts terminated by the foal (76.62±0.90 s vs. 77.24±0.94 s, n. s.; see Fig. 1). We found no general correlation between suckling bout duration and frequency (partial r_s_ = −0.03, n.s., with age of the foal eliminated).

Birth weight of the foals born within the season following observations of suckling behaviour did not differ in foals of lactating (66.2±1.01 kg) and non-lactating mares (64.4±1.04 kg, F_(1, 72)_ = 1.55, P = 0.22).

## Discussion

We found that domestic horse mares in some aspects altered their nursing behaviour during period of intensive lactation according to whether they were pregnant or not. Contrary to our predictions, however, we haven't found any signs of maternal care shortening towards nursed foals in pregnant mares which would be expected considering concurrent investment of a female into two offspring, a nursed offspring and a foetus; we found quite the opposite.

Pregnant mares did not either reject the foals' attempts to suck nor have they terminated suckling bout more often than non-pregnant nursing females, indicating no conflict between mother and offspring over suckling controlled by pregnancy. Considering negligible amount of suckling solicitation the mothers rejected (1% from suckling solicitations of the foals) it was predominantly through termination of suckling bouts (15% from successful suckling bouts) how the mares controlled the amount of maternal resources they provided to their nursed young. As expected, suckling bouts terminated by the mother were considerably shorter than those the foal stopped itself [15], [20]. We found that pregnant mare allowed her foal to suck longer when it was her who determined the length of a suckling bout. While frequency of suckling did not change in relation to pregnancy it means pregnant mares did not restrict, but increased filling of foal's needs compared to non-pregnant herdmates.

It suggests that lactating pregnant mares enhanced maternal investment to a nursed foal during early stages of pregnancy. This is most likely because they expect increasing trade-off between investment to a nursed foal and a growing foetus later in pregnancy when demands of their foetus increase. Low initial demands of a foetus increase from mid-gestation and most of the foetal growth during the last trimester of pregnancy [26]; pregnancy in horses normally ranges between 315 to 365 days, with the average being about 340 days [27]. This increase seems to be well reflected by equid mares who wean their foals earlier than barren ones [6], [10], [11], [13] and time the weaning often just around forthcoming of the last trimester of pregnancy.

Pregnant mares can presumably start altering their maternal behaviour towards a nursed foal early after conception as the recognition of pregnancy by the mare's organism should occur on physiological basis between day 7 and day 17 (when the constant movement of the equine conceptus throughout the uterus ensures release of maternal recognition of pregnancy signal onto the endometrium in all parts of the uterus [28].

Thus pregnant mares may, through more intensive maternal care (relaxed mother-offspring conflict) during early stages of lactation, compensate their nursed foals for later decrease in investment due to increasing demands of the foetus and/or for the shorter period of milk supply. Barren mares, on the other side, are not under pressure of dual investment and wean their foals usually later so they can spread their maternal investment over a longer period of lactation. In our study weaning process could not be investigated as foals were artificially weaned before they reached 7 months of age, all of them earlier than spontaneous weaning occurred or the mothers reached last trimester of pregnancy. However, as milk production declines considerably by six months of lactation [29], our observations covered the period of most intensive milk provision. Further research is needed in this area.

We did not detect any effect of mother's pregnancy on foals' frequency of suckling solicitations or duration of suckling bout stopped by the foal on its own which does not suggest effort of the foals to compensate for increased mother-offspring conflict in pregnant mares.

Low rates of either foals' suckling attempts rejected (1%) or sucklings terminated (15%) by the mother in our study most likely resulted from optimal nutrition the mares were provided with. It presumably means the foals' needs were fulfilled as suggested also in Estep et al. (1993). Since pregnancy of the mother apparently altered suckling behaviour under these conditions indicating a low level of parent-offspring conflict it should be considered further when analyzing suckling behaviour in terms of maternal investment or mother-offspring conflict in free-living population of equids exhibiting usually high mother-offspring conflict.

The only study providing evidence about effect of pregnancy on suckling characteristics during period of milk provision in equids [15] revealed just opposite trends to ours; pregnant captive plains zebra mares kept in ZOO allowed shorter suckling bouts to a foal when they themselves determined a length of a suckling bout which indicated higher parent–offspring conflict in pregnant mares compared to non-pregnant ones. In spite of this, results of both studies may not be in conflict due to different period the foals were investigated. While plains zebras study covered phase of weaning conflict as most of the foals were observed until natural weaning which occurred in pregnant mares from 243 to 355 days of foal's age [15], [18], all of our foals were artificially weaned before they reached the age of 210 days, i.e., mostly earlier than weaning process could have even started. Thus both studies are not comparable in this aspect and detailed analyses of mother-offspring conflict during particular stages of pregnancy/lactation should be performed.

Nursing a foal during the first two trimesters of pregnancy had no negative impact on birth weight of the foetus. However, the effect of lactation in later stages of pregnancy should be address to further research.

To our knowledge, this is the first study showing a lower mother-offspring conflict in pregnant than in non-pregnant lactating females while expecting just the opposite. Our results suggest that pregnant mares cope with parallel investment into a nursed foal and a foetus through enhancing nursing behaviour in early stages of pregnancy before the initially low requirements of the foetus increase. They compensate their suckling foal with the perspective of its early weaning due to ongoing pregnancy. We expect such role of pregnancy not just in free-living populations of equids, but also in other ungulate species with dual maternal investment, e.g., in cattle, buffalo or goats.
